# The surgical interval between robot-assisted SEEG and epilepsy resection surgery is an influencing factor of SSI

**DOI:** 10.1186/s13756-024-01438-w

**Published:** 2024-07-26

**Authors:** Xiaolian Xie, Hongwu Yao, Hulin Zhao, Bowei Liu, Yanling Bai, Huan Li, Yunxi Liu, Mingmei Du

**Affiliations:** 1 Department of Infection Management and Disease Control, Chinese PLA General Hospital, The 1st Medical Center, Fuxing Road No. 28, Beijing, 100853 China; 2 Central Sterile Supply Department, Ningxia People’s Armed Police Corps Hospital, South Qinghe Street No. 895, Yinchuan, 750001 China; 3 Department of Neurosurgery, Chinese PLA General Hospital, The 1st Medical Center, Fuxing Road No. 28, Beijing, 100853 China

**Keywords:** Stereoelectroencephalography, Radiofrequency thermocoagulation, Epilepsy resection surgery, Surgical site infection

## Abstract

**Background:**

In recent years, the development of robotic neurosurgery has brought many benefits to patients, but there are few studies on the occurrence of surgical site infection (SSI) after robot-assisted stereoelectroencephalography (SEEG). The purpose of this study was to collect relevant data from robot-assisted SEEG over the past ten years and to analyze the influencing factors and economic burden of surgical site infection.

**Methods:**

Basic and surgical information was collected for all patients who underwent robot-assisted SEEG from January 2014 to December 2023. Logistic regression was used to analyze the factors influencing SSI according to different subgroups (radiofrequency thermocoagulation or epilepsy resection surgery).

**Results:**

A total of 242 subjects were included in this study. The risk of SSI in the epilepsy resection surgery group (18.1%) was 3.5 times greater than that in the radiofrequency thermocoagulation group (5.1%) (OR 3.49, 95% CI 1.39 to 9.05); this difference was statistically significant. SSI rates in the epilepsy resection surgery group were associated with shorter surgical intervals (≤ 9 days) and higher BMI (≥ 23 kg/m^2^) (6.1 and 5.2 times greater than those in the control group, respectively). Hypertension and admission to the intensive care unit (ICU) were risk factors for SSI in the radiofrequency thermocoagulation group. Patients with SSIs had $21,231 more total hospital costs, a 7-day longer hospital stay, and an 8-day longer postoperative hospital stay than patients without SSI.

**Conclusions:**

The incidence of SSI in patients undergoing epilepsy resection after stereoelectroencephalography was higher than that in patients undergoing radiofrequency thermocoagulation. For patients undergoing epilepsy resection surgery, prolonging the interval between stereoelectroencephalography and epilepsy resection surgery can reduce the risk of SSI; At the same time, for patients receiving radiofrequency thermocoagulation treatment, it is not recommended to enter the ICU for short-term observation if the condition permits.

**Supplementary Information:**

The online version contains supplementary material available at 10.1186/s13756-024-01438-w.

## Introduction

Stereoelectroencephalogram (SEEG) [1] is a neurosurgical diagnostic technique for patients with drug-refractory focal epilepsy. When non-invasive preoperative evaluation is inconsistent or the surgical resection margin is not clear, it is used to determine the seizure area. SEEG allows electrodes to be implanted deep into the brain through small percutaneous drilling without the need for craniotomy. The position of the electrodes and the path through the brain are planned preoperatively based on the working hypothesis of the epileptogenic zone (EZ) location [2]. In general, SEEG can better research anatomic-electricityclinical seizures, epileptogenic zone in order to assist doctors better clear scop and achieving accurate epilepsy resection or SEEG-guided radiofrequency thermocoagulation (RF-TC) [3]. One of the most common indications for RF-TC involves the need for SEEG to determine whether surgery is feasible. RF-TC may be sufficient to cure patients when the epileptic seizure area is small enough to be completely covered by the sum of the coagulation volume [4].

Since the first robot-assisted SEEG surgery was successfully performed [5, 6], robotic surgery has been proven to be an efficient and safe technique that not only greatly reduces the duration of surgery and anesthesia but also has high accuracy [7]. Although robot-assisted SEEG surgery has provided many benefits in neurosurgery, surgical site infection (SSI) is still a difficult problem in nosocomial infection that needs to be considered. According to the literature, the incidence of SSI during neurosurgery fluctuates between 1 and 20% [8–10], which seriously increases the medical costs and family burdens of patients [11].

As various centers accumulate experience with robot-assisted SEEG monitoring, the goal of minimizing SSI, such as postoperative intracranial infections, moves to the forefront. However, studies on SSI after SEEG-guided RF-TC or epilepsy resection surgery are rare. Therefore, the main purpose of this report was to collect data on robot-assisted SEEG surgery over the past ten years and analyze the current status of SSI and its influencing factors. The secondary endpoint was to determine the increased financial burden associated with SSIs.

## Materials and methods

### Materials

We reviewed all patients who underwent robot-assisted SEEG in neurosurgery between January 1, 2014 to December 31, 2023, a total of 242 patients in a tertiary hospital in Beijing. Twenty-six of them suffered from SSI. All patients were divided into two groups according to the different epilepsy treatment techniques used (RF-TC or epilepsy resection surgery). The RF-TC group and the epilepsy resection surgery group were named group 1 and group 2, respectively. This study was approved by the Medical Ethics Committee (NO:S2019-142–02).

#### Inclusion criteria SEEG

The inclusion criteria for individuals were as follows: (1) were diagnosed with drug-resistant epilepsy (the patient has failed to achieve sustained seizure freedom despite adequate trials of two tolerated and appropriately chosen and used antiepileptic drugs schedules[12]); (2) underwent robot-assisted SEEG; (3) underwent RF-TC or epilepsy resection surgery; and (4) had complete case data.

#### Exclusion criteria

The exclusion criteria for patients were as follows: (1) underwent conventional frame SEEG surgery; (2) had electrodes removed after SEEG without RF-TC or epilepsy resection surgery; and (3) had incomplete data.

#### Diagnostic criteria for SSI

We defined SSIs within 90 days of surgery as intracranial infections according to the Centers for Disease Control and Prevention criteria[13]. The infections involved in this study are all intracranial infections. The diagnosis must meet the following criteria (1) and (2), and may comply with (3) or (4): (1) Patient has at least two of the following signs or symptoms: headache, dizziness, fever (> 38.0℃), localizing neurologic signs, changing level of consciousness, or confusion; (2) have increased white cells, elevated protein, and decreased glucose in cerebrospinal fluid; (3) have at least one of purulent drainage from the organ/space detected by gross anatomical exam or histopathologic exam or imaging test evidence definitive or equivocal for infection; (4) Patient has organism(s) identified from brain tissue or dura by a culture or non-culture based microbiologic testing method.

### Methods

The "real-time surgical site infection surveillance mode (SSISM)" was used to collect case information from patients with SSIs during robot-assisted SEEG surgery during the research period. Early warning and monitoring strategies for SSI in SSISM include postoperative body temperature, positive microbiological tests, and the increased use of antibiotics. These strategies can not only provide real-time warnings for all SSIs during hospitalization but also provide early warning signs for patients with suspected infections who are readmitted within 30 days after surgery. After the SSI warning information is generated, it will be directly pushed to infection control professionals and clinicians, and the SSI database will be generated after infection control professionals and clinicians jointly confirm the information[14].

#### RF-TC

RF-TC was performed after more than three habitual seizures were recorded. Before commencing the thermocoagulation process, a small electrical current is applied through the needle to ensure accurate positioning and avoid damage to surrounding tissues. Once the correct position is confirmed, radiofrequency energy is delivered through the needle, gradually increasing the temperature to the desired level. This thermal effect causes coagulation and denaturation of the nerve tissue, disrupting pain signals or abnormal neural activity[15].

### Statistical analysis

For bivariate analyses, we used the Pearson χ2 test for categorical variables and the Wilcoxon and Mann‒Whitney tests for nonparametric distributions. To identify independent risk factors for robot-assisted SEEG surgery SSI, we included variables found to be statistically significant in the crude analysis (P < 0.05) in the logistic regression model to estimate the odds ratios (ORs) and 95% confidence intervals (CIs) or p values. Subgroup analysis was performed according to the logistic regression model results. In the subgroup analysis, the optimal cutoff values of continuous variables were obtained by using restricted cubic bar plots, and patients were divided into high-risk and low-risk groups according to the cutoff values. A logistic regression model was subsequently used to determine whether the SSI distributions differed between the low-risk and high-risk groups. Two-sided p values less than 0.05 were considered significant. All the statistical analyses were performed using R version 4.3.2.

## Results

A total of 242 epilepsy patients who underwent robot-assisted SEEG were included. Three cases were excluded, including 2 cases of conventional framework SEEG surgery, and 1 case could not receive follow-up treatment ( RT-TC or resection) due to infection after electrode implantation and monitoring failure of epileptic area. The median age of the patients was 22.5 years (range: 1–54), and the majority of patients were male (174, 71.9%). Approximately 10.7% of the included patients had hypertension. The median duration of surgery was 2.08 h (range: 1.58–2.67). Among the 242 patients, 84 had a rosa robot, and 158 had a remobot (34.7% vs. 65.3%, respectively). Among those included, 105 patients underwent epilepsy resection surgery, while 137 patients were treated with RF-TC.

### Prevalence of SSIs and influencing factors

In our study, 26 of 242 (10.74%) patients experienced SSI. All 26 SSIs were intracranial infections. A comparison of the prevalence and distribution of SSIs among patients between 2014 and 2023 is presented in Table 1. A total of 19 of the 105 patients (18.1%) who underwent epilepsy resection surgery had SSIs, whereas 7 of the 137 patients (5.1%) who underwent RF-TC had SSIs (P < 0.05). Moreover, there were no significant differences in terms of median age at surgery, length of hospital stay before surgery or robot type.

**Table 1:** The comparison of variables of 242 patients with or without SSIs

Characteristic	ALL patients (N = 242)	Patients without SSI (N = 216)	Patients with SSI (N = 26)	*Z* value	*P* value
Age at surgery (IQR)	23 (12, 32)	21 (12, 31)	25.5 (18, 35)	− 1.83	0.067
Age (%)				4.17	**0.041**
≤ 18 yr	101 (41.7)	95 (94.1)	6 (5.9)		
> 18 yr	141 (58.3)	121 (85.8)	20 (14.2)		
Gender (%)				3.95	**0.047**
Male	174 (71.9)	151 (86.8)	23 (13.2)		
Female	68 (28.1)	65 (95.6)	3 (4.4)		
BMI(kg/m^2^) (IQR)	22.17 (19.18, 25.82)	21.78 (18.85, 25.50)	25.13 (22.79, 27.17)	− 3.07	**0.002**
Hypertension (%)				4.62	**0.044**
No	216 (89.3)	196 (90.7)	20 (9.3)		
Yes	26 (10.7)	20 (76.9)	6 (23.1)		
Length of hospital stay before surgery (IQR)	10 (7, 14)	10 (7, 14)	11 (7, 14)	− 0.08	0.937
Robot type (%)				3.01	0.083
Rosa	84 (34.7)	71 (84.5)	13 (15.5)		
Remrobot	158 (65.3)	145 (91.8)	13 (8.2)		
NNIS Risk Index (%)				8.67	**0.003**
0	186 (76.9)	172 (92.5)	14 (7.5)		
1	56 (23.1)	44 (78.6)	12 (21.4)		
Number of electrodes(IQR)	8 (6, 10)	8 (6, 10)	7 (5, 8)	− 2.87	**0.004**
Admission to the ICU (%)				10.45	**0.001**
No	137 (56.6)	130 (94.9)	7 (5.1)		
Yes	105 (43.4)	86 (81.9)	19 (18.1)		
Epilepsy treatment techniques (%)				10.45	**0.001**
RF-TC	137 (56.6)	130 (94.9)	7 (5.1)		
Epilepsy Resection Surgery	105 (43.4)	86 (81.9)	19 (18.1)		

SSI, surgical site infection; CI, confidence interval; IQR, interquartile rangeAbbreviations; BMI, body mass index;

NNIS, National Nosocomial Infections Surveillance; ICU, Intensive Care Unit; RF-TC, Radiofrequency Thermocoagulation

A multivariate logistic regression model revealed that SSI was related to epilepsy treatment technique, BMI and higher NNIS risk index score. Epilepsy treatment technique is an important variable, and the incidence of SSI in the epilepsy resection surgery group was 3.5 times greater than that in the RF-TC group (OR 3.49, 95% CI 1.39 to 9.05).

### Factors influencing SSI in different patient subgroups

**Fig. 1:**
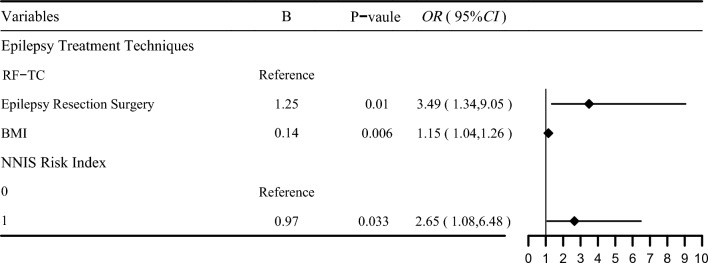
Forest plot illustrating the results of multivariate analysis for risk factors of SSI

The forest plot in Fig. 1 shows that epilepsy treatment technique is an important variable affecting the incidence of SSI. Therefore, we performed subgroup analyses according to epilepsy treatment technique.

Variables affecting infected patients with RF-TC are listed in Table 2. No significant difference in the number of electrodes implanted was observed between patients with and without SSI, while the median operation time varied significantly between the two groups; the SSI group had a longer operation time (2.58 (1.83, 2.92) hours vs. 1.78 (1.4, 2.35) hours, p = 0.03). Univariate analysis of risk factors revealed that hypertension, blood loss, operation time, surgical doctor status and longer ICU stays were significantly associated with SSI. Furthermore, the multivariate analysis shown in Table 2 revealed that the independent risk factors for SSI following RF-TC were hypertension and admission to the ICU. The median ICU stay for RF-TC patients was 1 day, with the majority remaining 1 day and one patient staying 15 days due to intracranial infection with lower respiratory tract infection and bacteremia (Table 3).

**Table 2:** Results of univariate analysis in different subgroups

Characteristic	RF-TC (Group 1)		Epilepsy Resection Surgery (Group 2)	
	Patients without SSI	Patients with SSI	Patients without SSI	Patients with SSI
Age (%)				
≤ 18 yr	71 (97.3)	2 (2.7)	24 (85.7)	4 (14.3)
> 18 yr	59 (92.2)	5 (7.8)	62 (80.5)	15 (19.5)
Gender (%)				
Male	82 (92.1)	7 (7.9)	69 (81.2)	16 (18.8)
Female	48 (100.0)	0 (0.0)	17 (85.0)	3 (15.0)
BMI(kg/m^2^) (IQR)	21.45 (18.32,25.54)	24.49 (20.98,28.08)	22.28 (19.18, 25.52)	25.35 (23.44, 26.81^*^
Hypertension (%)				
No	120 (96.8)	4 (3.2)^*^	76 (82.6)	16 (17.4)
Yes	10 (76.9)	3 (23.1)	10 (76.9)	3 (23.1)
Length of hospital stay before surgery@(IQR)	10 (7, 13.25)	11 (7, 15)	12 (8, 15)	11 (7, 14)
Operation time@(hour) (IQR)	0.45 (0.31, 0.65)	0.49 (0.33, 0.70)	4.67 (4, 5.565)	4.96 (4.17, 6.0025)
Number of electrodes (IQR)	9.5 (8, 11)	10 (7, 10)	6 (5, 8)	6 (5, 7)
Blood loss(ml) (IQR)	0^#^	0^#^	200 (100, 300)	200 (150, 300)
NNIS Risk Index (%)				
0	111 (95.7)	5 (4.3)	61 (87.1)	9 (12.9)
1	19 (90.5)	2 (9.5)	25 (71.4)	10 (28.6)
Surgery Doctor(IQR)				
Doctor A	21 (80.8)	5 (19.2)^*^	22 (71.0)	9 (29.0)
Doctor B	100 (98.0)	2 (2.0)	7 (100.0)	0 (0.0)
Doctor C	NA	NA	33 (84.6)	6 (15.4)
Other Doctor	9 (100.0)	0 (0.0)	24 (85.7)	4 (14.3)
Admission to the ICU (%)				
No	118 (96.7)	4 (3.3)^*^	12 (80.0)	3 (20.0)
Yes	12 (80.0)	3 (20.0)	74 (82.2)	16 (17.8)
The interval between two procedures@(day)(IQR)	NA	NA	9.5 (6, 12.25)	7 (6, 9)^*^

P values <0.05 in bold indicate that the difference between infected and uninfected patients is statistically significant

^*^The difference between infected patients and uninfected patients was statistically significant (P Vaule < 0.05); ^#^, There was no bleeding in the whole process of RF-TC, only one case had local adhesion after thermocoagulation, and a small amount of bleeding occurred during the removal of the electrode; NA, not applicable

**Table 3:** Results of multivariate analysis in different subgroups

Different Groups		B	SE	Wald	P Vaule	OR Vaule	95%CI	
							Lower	Upper
Group 1	Intercept	− 4.13	0.73	31.93	** < 0.001**	0.01		
	Hypertension							
	No	Reference						
	Yes	2.67	0.97	7.59	**0.006**	14.49	2.16	97.15
	Admission to the ICU							
	No	Reference						
	Yes	2.49	0.97	6.65	**0.01**	12.02	1.82	79.64
Group 2	Intercept	− 3.39	1.85	3.38	**0.066**	0.03		
	BMI							
	≤ 23 kg/m^2^	Reference						
	> 23 kg/m^2^	1.64	0.63	6.92	**0.009**	5.17	1.52	17.60
	Surgery Interval							
	> 9 days	Reference						
	≤ 9 days	1.81	0.69	6.96	**0.008**	6.10	1.59	23.33

The numbers in bold (P <0.05) indicate that the variable was significant in the multivariate analysis

CI, confidence interval

**Fig. 2:**
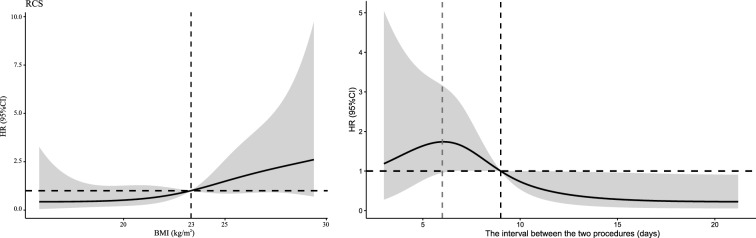
Restricted cubic splines for SSI according to BMI and surgery interval

Univariate analysis of the epilepsy resection surgery group revealed statistically significant differences in BMI and the interval between the two surgeries between infected and uninfected patients. In Fig. 2, we used restricted cubic splines to flexibly model and visualize the relationships of the predicted BMI and surgical interval with SSI. SSI was nonlinearly related to BMI, with a stepwise increase in the risk of SSI occurring when the BMI was greater than 23 kg/m^2^. The RCSs of the SSIs and surgical intervals showed that the risk of SSIs increased gradually before the 6th day and decreased gradually after the 6th day. The hazard ratio (HR) was 1 at 9 days, which indicated that a duration less than or equal to 9 days was a risk factor for SSI, and a duration greater than 9 days was a protective factor against SSI. Therefore, we divided patients into high-risk and low-risk groups based on BMI = 23 kg/m^2^ and surgery interval = 9 days, respectively, and simultaneously included multivariate logistic regression for risk factor analysis; both variables were statistically significant. A high BMI and operative time less than or equal to 9 days were risk factors for SSI in the epilepsy resection surgery group. When the surgery interval was ≤ 9 days, the risk of SSI increased by 6 times (OR = 6.10, 95% CI 1.59 to 23.33).

### Economic burden of SSI

Table 4 shows the different hospitalization costs and total costs. Patients with SSIs had substantially greater total median costs ($21,231) (IQR: 17,666–24,877) than did those without SSIs ($17,033) (IQR: 13,982–20,692). There was an additional $4,198 in costs for the infection group compared to the noninfection group (p = 0.001). These costs were largely driven by medication costs in the infection group.

**Table 4:** Economic burden of SSI

Variables	All Patients (N = 242)		Z value	P value
	Patients without SSI (N = 216)	Patients with SSI (N = 26)		
Total Costs($) (IQR)	17,033 (13,982, 20,692)	21,231 (17,666, 24,877)	− 3.36	**0.001**
Bed Fee($) (IQR)	213 (160, 280)	294 (254, 404)	− 4.50	** < 0.001**
Radiation Fee($) (IQR)	40 (31, 84)	48 (41, 80)	− 1.71	0.088
Nursing Fee($) (IQR)	78 (52, 119)	149 (113, 180)	− 4.64	** < 0.001**
Laboratory Fee($) (IQR)	310 (211, 561)	932 (605, 1329)	− 6.08	** < 0.001**
Surgical Fee($) (IQR)	13,695 (11,635, 15,498)	13,051 (11,976, 15,673)	− 0.04	0.966
Special examination Fee($) (IQR)	615 (354, 1043)	656 (466, 908)	− 0.66	0.507
Medication Fee($) (IQR)	663 (272, 1999)	3,763 (2,560, 5,241)	− 6.53	** < 0.001**
Diagnostic Fee($) (IQR)	336 (238, 434)	476 (378, 644)	− 4.34	** < 0.001**
Therapy Fee($) (IQR)	262 (159, 864)	1,045 (832, 1,475)	− 5.35	** < 0.001**
Other expenses($) (IQR)	29 (19, 45)	37 (27, 62)	− 2.32	**0.02**
Days of hospitalization (day)(IQR)	26 (19, 33)	33 (27, 40)	− 3.64	** < 0.001**
Postoperative length of hospital stay(day)(IQR)	13 (9, 19)	21 (19, 28)	− 5.48	** < 0.001**

P values <0.05 in bold indicate that the difference between infected and uninfected patients is statistically significant

The overall cost of the infection group increased (1.2 times greater than that of the noninfection group), and when stratified into different project costs, the cost of medicine increased the most, followed by the cost of other treatments and laboratory tests (5.7, 4.0 and 3.0 times greater than that of the noninfection group, respectively).

Compared with surgical patients without SSI, patients with SSI had an increased overall hospital stay of 7 days and an increased postoperative hospital stay of 8 days.

## Discussion

SSI can occur after robot-assisted SEEG mainly because patients underwent epilepsy resection surgery or RF-TC. SSI after electrode placement is rare (0.4%, 1/243), and the reported incidence in the literature is 0.0% ~ 0.8% [16, 17], which is consistent with the findings of this study.In our study, the risk of SSI after epilepsy resection surgery (18.1%) was 3.5 times greater than that after RF-TC (5.1%). The SSI rate after epilepsy resection surgery was 18.1%, and the infection rate reported by other scholars was 9.6% [18]. The infection rate during epilepsy resection surgery in this study was high, mainly because all patients underwent robot-assisted SEEG before craniotomy, and the literature research results showed that the risk of SSI in patients undergoing EEG monitoring before resection surgery (9.6% vs 2.4%) increased fourfold (OR 4.32, 95% CI 2.1 to 8.9; p < 0.001)[18]. SEEG destroys the integrity of the scalp barrier and easy to cause bacterial reproduction or colonization. Moreover, our study revealed that the risk of SSI increased by 6 times when the operation interval between SEEG and epilepsy resection surgery was ≤ 9 days. A shorter operation interval is not conducive to the recovery of patient immunity. Therefore, prolonging the surgical interval between two surgeries can effectively reduce the incidence of SSI during epilepsy resection surgery.

The SSI rate in patients who underwent SEEG-guided RF-TC was 5.1%. This result was similar to that of a study in which 199 patients were eligible for analysis and 8 (4.0%) developed SSIs [19]. The risk of SSI in the epilepsy resection group was 3.5 times greater than that in the RF-TC group. When surgical resection is not superior to RF ablation for improving epilepsy symptoms or when seizures are nonfocal or multifocal, RF-TC is recommended as the preferred treatment so that patients can at least avoid unnecessary craniotomy[20, 21]. Interestingly, in our study, we found that the number of electrodes implanted was significantly lower in the epilepsy resection surgery group than in the RF-TC group. We used restricted cubic bar plots to analyze the relationship between the number of implanted electrodes and the choice of epilepsy resection surgery (the figure in the supplementary information) and found that when the number of electrodes was less than 8, it was easier to choose epilepsy resection surgery. In summary, when the number of electrodes implanted is low, the chance of resection is greater. However, when we increase the number of electrode implantations to 8 or more, whether it will expand probability of cure of RF-TC avoiding the resection requires further study. Meanwhile, the increasing cost of surgery caused by the increase in the number of electrodes still needs our attention.

A higher BMI (especially when the BMI is greater than 23 kg/m^2^) in the epilepsy resection surgery group was associated with the incidence of SSI, consistent with what has been shown in the literature [22]. However, no association was found between BMI and SSI in the SEEG-guided RF-TC group. We found that the median age of patients in the epilepsy resection surgery group (median age 25 years, range 18–32 years) was older than that of patients in the RF-TC group (median age 16 years, range 9–31 years); moreover, both groups were adults, indicating that BMI may be related only to SSI during adult epilepsy resection surgery and that its correlation with SSI in children needs further study.

Comorbid illnesses could predispose patients to SSI acquisition [23]. Our study revealed that hypertension was found in 23.1% of the patients with SSIs. In accordance with these findings, a study in Saudi Arabia showed that 62.5% of patients with SSIs had hypertension [24]. However, it is worth noting that hypertension was an influencing factor of SSI in only the SEEG-guided RF-TC group and was not related to SSI after epilepsy resection surgery.

A preoperative stay in an intensive care unit (ICU) is associated with an increased risk of SSI. Patients in ICUs are twice as likely to face SSI than are those in general wards [25]. We observed that SSIs occurred in 20% of the RF-TC patients admitted to ICUs, and the median length of stay in the ICUs was 1 day. Patients admitted to ICUs are susceptible to infection due to their low immune status and various invasive procedures. In addition, more bacteria in the ICU environment are more likely to cause cross-infection between patients.

Thus, to reduce the incidence of SSI, patients receiving SEEG-guided RF-TC should focus on patients with chronic diseases such as hypertension. If the patient’s condition permits, it is not recommended that she enter the ICU after surgery.

We also found that the SSI rate of SEEG-guided RF-TC according to single factor analysis was related to the surgeon (doctor A, 19.2%; doctor B, 2%), but there was no significant difference in multiple factors. This may be due to the small sample size of our study. It is suggested that the analysis be carried out again when more cases are accumulated in the later stage.

SSIs account for more than A$323.5 million in health care costs in Australia each year [26]. In our study, the total hospitalization cost of infected patients increased by $4198, of which the three costs associated with the greatest increase were drug costs, treatment costs and laboratory examination costs, and there was no significant increase in surgery, radiation or other costs. This work demonstrated that infection is related to substantially increased costs to the healthcare system compared to those who did not develop an infection. Compared with SSI-free patients, SSI patients had longer hospital stays (7 to 8 days), which is consistent with the findings of an Australian Public Hospital study of 16,541 SSIs (8 days, 95% CI 7.9–8.1) [26] and indirectly increased additional medical expenses.

It is worth noting that this study has the following limitations. First, the number of patients included is limited and is conducted in a single center. Therefore, in order to draw a clear conclusion on the universality of the data, it is necessary to carry out a larger multicenter study. Second, this study did not follow up all patients and may have lost some SSI patients. However, studies have shown that the effective rate of our hospital infection real-time monitoring system for monitoring SSI in neurosurgery is 93.02% [14]. Third, compared with robot surgery, traditional stereotactic surgery has positioning blind spots, which is rarely performed after the introduction of robots, resulting in a small sample size. Therefore, this paper does not compare traditional stereotactic SEEG with robot-based SEEG surgery. Fourth, the treatment effect of epilepsy was not followed up and the related factors of surgical technique were not studied, which was suggested to be increased in future studies. Fifth, because the indications of RF-TC are different from those of resection, the comparison of SSI may have certain limitations.

## Conclusion

The risk of SSI in the epilepsy resection group was 3.5 times greater than that in the RF-TC group. To reduce the incidence of SSI during resection surgery for epilepsy, the operation interval can be extended to more than 9 days. The SSI rate in patients who underwent SEEG-guided RF-TC was 5.1%, and the occurrence of infection was related to the patient's hypertension and entry into the ICU. It is not recommended that patients enter the ICUs for short-term observation after surgery if the patient’s condition permits. SSI increased the total cost of hospitalization by $4,198 and was associated with longer hospital stays than patients without SSI.

### Supplementary Information

Additional file1

## Data Availability

The datasets used and/or analyzed during the current study are available from the corresponding author upon reasonable request.

## Abbreviations

SEEG: Stereoelectroencephalography
EZ: Epileptogenic zone
RF-TC: Radiofrequency thermocoagulation
SSI: Surgical site infection
SSISM: Surgical site infection surveillance mode
CI: Confidence interval
IQR: Interquartile range
BMI: Body mass index;
NNIS: National nosocomial infections surveillance
ICU: Intensive care unit

## References

[1] Murray-Douglass A, Papacostas J, Ovington A, Wensley I, Campbell R, Gillinder L. Stereoelectroencephalography: a review of complications and outcomes in a new Australian centre. Intern Med J. 2024. 10.1111/imj.16284. 38165070 10.1111/imj.16284

[2] Li P, Zhou Y, Zhang Q, Yang Y, Wang M, Zhu R, et al. Frameless robot-assisted stereoelectroencephalography-guided radiofrequency: methodology, results, complications and stereotactic application accuracy in pediatric hypothalamic hamartomas. Front Neurol. 2023. 10.3389/fneur.2023.1259171. 37928157 10.3389/fneur.2023.1259171 PMC10621047

[3] Youngerman BE, Khan FA, McKhann GM. Stereoelectroencephalography in epilepsy, cognitive neurophysiology, and psychiatric disease: safety, efficacy, and place in therapy. Neuropsych Dis Treat. 2019;15:1701–16. 10.2147/NDT.S177804 PMC6610288 31303757

[4] Bourdillon P, Rheims S, Catenoix H, Montavont A, Ostrowsky-Coste K, Isnard J, et al. Surgical techniques: Stereoelectroencephalography-guided radiofrequency-thermocoagulat-ion (SEEG-guided RF-TC). Seizure. 2020;77:64–8. 30711397 10.1016/j.seizure.2019.01.021

[5] Zhang D, Cui X, Zheng J, Zhang S, Wang M, Lu W, et al. Neurosurgical robot-assistant stereoelectroencephalography system: Operability and accuracy. Brain Behav. 2021. 10.1002/brb3.2347. 34520631 10.1002/brb3.2347 PMC8553331

[6] Zhou S, Gao Y, Li R, Wang H, Zhang M, Guo Y, et al. Neurosurgical robots in China: State of the art and future prospect. iScience. 2023;26:107983. 10.1016/j.isci.2023.107983. 37867956 10.1016/j.isci.2023.107983 PMC10589856

[7] Philipp LR, Matias CM, Thalheimer S, Mehta SH, Sharan A, Wu C. Robot-assisted Stereotaxy reduces target error: a meta-analysis and meta-regression of 6056 trajectories. Neurosurgery. 2021;88:222–33. 33045739 10.1093/neuros/nyaa428

[8] Yasuhara T, Date I. Surgical site infection(SSI)in neurosurgery. No Shinkei Geka. 2021;49:1093–104. 34615769 10.11477/mf.1436204493

[9] Han C, Song Q, Ren Y, Luo J, Jiang X, Hu D. Dose-response association of operative time and surgical site infection in neurosurgery patients: a systematic review and meta-analysis. Am J Infect Control. 2019;47:1393–6. 31296347 10.1016/j.ajic.2019.05.025

[10] Gu Z, Wang Q, Chen J, Zhu Y. Predicted factors of surgical site infection in glioblastoma patients: a meta-analysis. Int Wound J. 2023. 10.1111/iwj.14504. 38044279 10.1111/iwj.14504 PMC10898386

[11] NIHR Global Research Health Unit on Global Surgery. Routine sterile glove and instrument change at the time of abdominal wound closure to prevent surgical site infection (ChEETAh): a pragmatic, cluster-randomized trial in seven low-income and middle-income countries. Lancet. 2022;400(10365):1767–76. 36328045 10.1016/S0140-6736(22)01884-0

[12] Kwan P, Arzimanoglou A, Berg AT, Brodie MJ, Allen Hauser W, Mathern G, et al. Definition of drug resistant epilepsy: consensus proposal by the ad hoc task force of the ILAE Commission on Therapeutic Strategies. Epilepsia. 2010;51(6):1069–77. 19889013 10.1111/j.1528-1167.2009.02397.x

[13] National Healthcare Safety Network, Centers for Disease Control and Prevention. Surgical site infection (SSI) event. http://www.cdc.gov/nhsn/pdfs/pscmanual/9pscssicurrent.pdf. Published January 2017. Accessed January 25, 2017

[14] Du M, Li M, Liu K, Suo J, Xing Y, Liu B, et al. A real-time surgical site infections surveillance mode to monitor surgery classification specific, hospital-wide surgical site infections in a Chinese tertiary hospital. Am J Infect Control. 2017;45:430–2. 28185667 10.1016/j.ajic.2016.12.002

[15] Wang L, Jin W, Zhang Y, Wang S, Li Q, Qin J, et al. Stereoelectroencephalography-guided radiofrequency thermocoagulation in drug-resistant focal epilepsy. Ann Transl Med. 2022. 10.21037/atm-21-6851. 35280357 10.21037/atm-21-6851 PMC8908190

[16] Ho AL, Feng AY, Kim LH, Pendharkar AV, Sussman ES, Halpern CH, et al. Stereoelectroencephalography in children: a review. Neurosurg Focus. 2018. 10.3171/2018.6.FOCUS18226. 30173607 10.3171/2018.6.FOCUS18226

[17] Chaitanya G, Romeo AK, Ilyas A. Robot-assisted stereoelectroencephalography exploration of the limbic thalamus in human focal epilepsy: implantation technique and complications in the first 24 patients. Neurosurg Focus. 2020. 10.3171/2020.1.FOCUS19887. 32234983 10.3171/2020.1.FOCUS19887

[18] Gooneratne IK, Mannan S, de Tisi J, Gonzalez JC, McEvoy AW, Miserocchi A, et al. Somatic complications of epilepsy surgery over 25 years at a single center. Epilepsy Res. 2017;132:70–7. 28324680 10.1016/j.eplepsyres.2017.02.016

[19] Meng Y, Voisin MR, Suppiah S, Merali Z, Moghaddamjou A, Alotaibi NM, et al. Risk factors for surgical site infection after intracranial electroencephalography monitoring for epilepsy in the pediatric population. J Neurosurg Pediatr. 2018;22:31–6. 29624147 10.3171/2018.1.PEDS17476

[20] Bourdillon P, Devaux B, Job-Chapron AS, Isnard J. SEEG-guided radiofrequency thermocoagulation. Neurophysiol Clin. 2018;48:59–64. 29273383 10.1016/j.neucli.2017.11.011

[21] Moles A, Guénot M, Rheims S, Berthiller J, Catenoix H, Montavont A, et al. SEEG-guided radiofrequency coagulation (SEEG-guided RF-TC) versus anterior temporal lobectomy (ATL) in temporal lobe epilepsy. J Neurol. 2018;265:1998–2004. 29943202 10.1007/s00415-018-8958-9

[22] Park H, de Virgilio C, Kim DY, Shover AL, Moazzez A. Effects of smoking and different BMI cutoff points on surgical site infection after elective open ventral hernia repair. Hernia. 2021;25:337–43. 32318887 10.1007/s10029-020-02190-x

[23] Yang J, Zhang X, Liang W. A retrospective analysis of factors affecting surgical site infection in orthopaedic patients. J Int Med Res. 2020;48(4):300060520907776. 32281431 10.1177/0300060520907776 PMC7155240

[24] AlGamdi SS, Alawi M, Bokhari R, Bajunaid K, Mukhtar A, Baeesa SS. Risk factors for surgical site infection following spinal surgery in Saudi Arabia: a retrospective case-control study. Med (Baltimore). 2021. 10.1097/MD.0000000000025567. 10.1097/MD.0000000000025567 PMC8084009 33907106

[25] Izadi N, Eshrati B, Mehrabi Y, Etemad K, Hashemi-Nazari SS. The national rate of intensive care units-acquired infections, one-year retrospective study in Iran. BMC Public Health. 2021. 10.1186/s12889-021-10639-6. 33781227 10.1186/s12889-021-10639-6 PMC8006501

[26] Royle R, Gillespie BM, Chaboyer W, Byrnes J, Nghiem S. The burden of surgical site infections in Australia: a cost-of-illness study. J Infect Public Health. 2023;16:792–8. 36963144 10.1016/j.jiph.2023.03.018

