# Supplementary material for: The surgical interval between robot-assisted SEEG and epilepsy resection surgery is an influencing factor of SSI
Source: Antimicrob Resist Infect Control. 2024 Jul 26;13:81. doi: 10.1186/s13756-024-01438-w (PMC11282661; doi:10.1186/s13756-024-01438-w)
Supplement: Supplementary file 1 — Additional file1 [file 13756_2024_1438_MOESM1_ESM.docx]

**Supplementary Information**

**
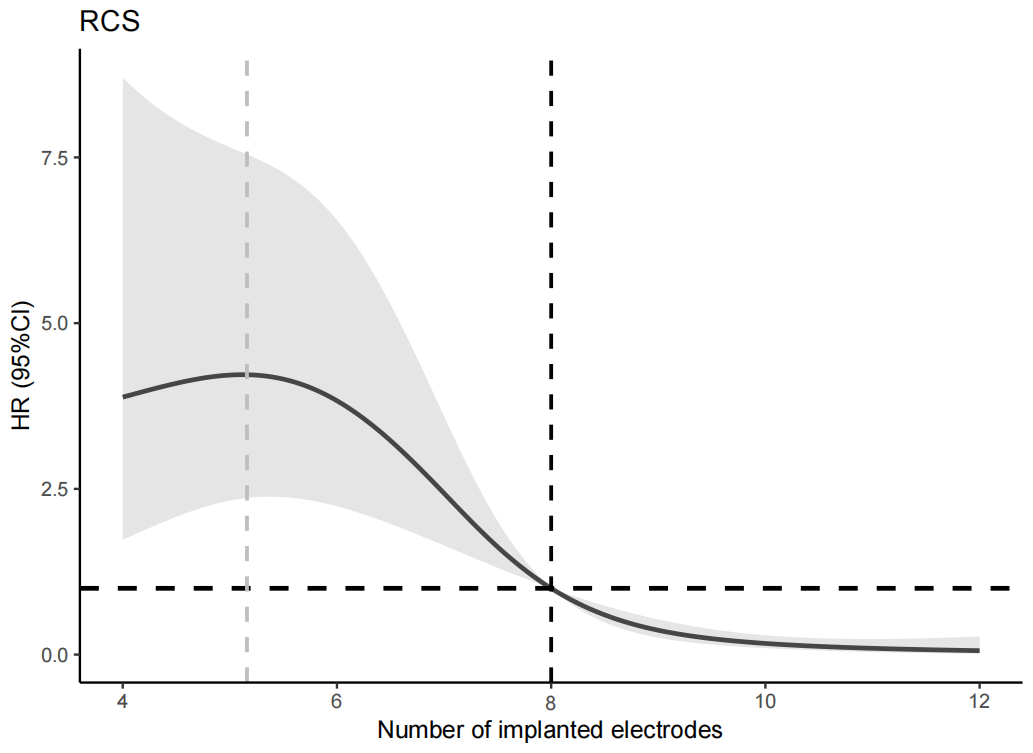
**

Figure 3. Restricted cubic splines of the relationship between the number of implanted electrodes and choice of epileptic resection surgery
